# Practical Medicine

**Published:** 1876-07

**Authors:** 


### II. PRACTICAL MEDICINE.

1. *The Hydrotherapeusis of Croup.* WINTERNITZ. (*Aester. Jahrb. für Paediatrik; Giorn. della R. Accad. di Medic.* Jan. 30, 1876.)

The author attributes great importance to the paretic condition of the dilator of the glottis as a cause of the distressing dyspnœa, and believes that to this, treatment should be specially directed.

Reflex excitation of the respiratory centre is therefore to be accomplished by mechanical or other methods. Cold affusion of water of various temperatures is to be practiced over the integument, and, if this is not sufficient, the author advises the use of the occipital douche. Four grave cases treated by this method are reported in full. W. invariably noticed, when employing the cold aspersion, that the force of the patient, well nigh spent, was reanimated, the cough was relieved, and the pulse and respiration became more tranquil. The remedy was specially valuable in preventing death by suffocation, which was the more frequent issue when the natural resolution of the disease was awaited.

2. *Pneumatic Treatment of Pulmonary Disease.* SCHNITZLER. (*Rev. des Sci. Méd.* Jan. 15, 1876.)

The treatment by compressed air considered to be indicated in the following pathological conditions: 1. General feebleness of the respiratory organs. 2. Chronic catarrh of the bronchi. 3. Pulmonary catarrh and the first stage of phthisis. 4. Emphysema, where this treatment is generally very efficacious. 5. In certain cases of nervous asthma, that is, in cases where a minute physical examination cannot detect any morbid changes in the heart or lungs. 6. Thus far S. has made few experiments in treating by this method affections of the larynx, because the dyspnœa is not the predominant symptom, as in a small number of disorders of this organ. There are, however, examples where the inspiration of compressed air has been of service in laryngeal disease.

The pneumatic method can be employed also in cases of stenosis of the larynx which are not extreme.

In diseases of the heart, the author recommends inspiration of compressed air and expiration in rarefied air, but the results thus far are not highly gratifying, and, at the best, there was but a transient amelioration of symptoms. Occasionally signs of cerebral congestion occurred (dizziness, ringing in the ears, cephalalgia, etc.,) which necessitates immediate abandonment of the treatment.

w

3. *Sedative Solution in Whooping-Cough.* GUÉNEAU DE MASSY. (*Jour. de Med. et de Chir. Pratiques.*)

Musk, three grains; potassic bromide, half a drachm to two scruples; cherry laurel water, one drachm and a half; syrup of ether, half an ounce; syrup of belladonna and syrup of codeine, of each, one ounce; syrup of orange flower, one ounce and a half. To a child eight to ten years of age, give a teaspoonful between meals, morning, evening and night. During the day it is not to be used, lest the narcotics recommended disturb digestion. The musk, if insupportable, may be omitted.

4. *Sleep of the Aged.* POPELAUER. (*Revue des Sciences Méd.* Jan. 15, 1876.)

Three forms: 1. *Continued sleep*, from which they must be aroused in order to eat, and to which they return immediately after. Appetite unchanged, obtuseness of intellect, loss of memory, prolonged sojourn in bed, gangrenous degeneration of skin. 2. *Diurnal sleep*, with agitation when night falls, inability to find the bed which has just been abandoned, getting into another bed than their own. This is the sleep at the onset of senile dementia. 3. *Agrypnia* with complete insomnia—the precursor of a real affection of the mind. This condition is to be treated by opium in large doses, which, as generally believed now, rarely induces dyspepsia, cephalic congestion or collapse.

5. *Milk Diet in the Albuminuria of Pregnancy.* TARNIER. (*Jour. de Med. et de Chir. Pratiques.*)

For several years the author has submitted pregnant and albuminuric women to a milk diet, and has, as invariably, noticed the albuminuria disappear and eclampsia never occur. The cure of the albuminuria of pregnancy thus made possible, leads him to hope that in a large proportion of cases eclampsia may be prevented by the same measures. In point of fact, eclampsia almost never occurs except in albuminuric women; and often suddenly appears before the onset of labor, the latter following convulsive attacks, but so soon after that the physician recognizes at the same time the symptoms of eclampsia and the signs of labor. Although rare cases of eclampsia without albuminuria occur, it might be said that if we were in position to cure the albuminuria of pregnancy we should at the same time possess the means of preventing eclampsia.

In order to do this, the albuminuria must be attacked in time. Unfortunately the latter is insidious in its invasion and is sometimes manifested by no peculiar symptoms. T., accordingly, frequently examines the urine of all pregnant women in his charge, and as soon as albuminuria declares itself, he puts them on a milk diet as follows: 1st day, one litre of milk, two portions of food; 2nd day, two litres of milk, one portion of food; 3rd day, three litres of milk and a half portion of food; 4th and following days, four litres of milk, or milk *ad libitum*, without other solid or liquid aliment.

In grave cases, if some of the prodromata of epilepsy have occurred, this gradation is not observed, and the patients are placed at once upon three or four litres of milk daily.

The influence of the milk diet is speedily manifested, and, in eight or fifteen days, after the commencement of treatment, the albuminuria is either greatly decreased or quite removed.

6. *Remarkable Case of Peritonitis.* E. J. ABBOTT, M.D. (*Amer. Prac.*, May, 1876.)

In this case, with no knowledge of the previous history, the patient was known to have died with symptoms of subacute peritonitis, with effusion.

Post mortem—the whole peritoneal surface was found covered with a layer of lymph a quarter of an inch in thickness. There were no adhesions of any consequence. "There was found just below the splenic flexure of the colon, an irregularly shaped piece of porcelain, evidently broken from a plate or dish." It was a fourth of an inch in thickness and must have required an opening in the intestine at least an inch and a quarter in length to allow of its escape. The opening of exit had entirely healed ; it could not be found.

7. *Diphtheria affecting the Nipples.* T. L. Wright, M.D. (*Cin. Lancet and Obs.*, May, 1876.)

This case is of a woman 28 years old, who, a few days after confinement, began to have sore nipples. Soon the milk was drawn with difficulty and was pinkish in color. In two weeks there appeared frequently in the milk fibrinous, stringy masses that seemed to be organized casts of the lacteal ducts. The stringy masses grew more frequent, the flow was more obstructed, and finally, in three weeks from the birth of the child, and after the breasts had become swollen and painful and threatened abscess, the nipples became covered with a thick, dense false membrane. This was dissected off carefully when it rapidly reformed. The milk was now allowed to "dry up," yet the hardness and swelling did not disappear, although the size was lessened. At the end of six months and after cancer had been feared, an abscess formed in the right breast and discharged for a week, afterward the breast became atrophied. "Associated with this fibroid condition of the right breast was a constant and severe pain in the right arm." While the difficulty was going on in the breast, there appeared an indolent swelling of the size of a hazlenut on the lower and back part of the radius. This afterward became an open sore, and discharged pus and pieces of bone. Dr. W. regarded the mammary affection as diphtheritic.

8. *A Case of Pica.* A. N. Gould, M.D. (*Boston Med. & Surg. Jour.*, April 13, 1876.)

An unmarried woman, 43 years old, had had generally through life good health. Menstruation had been normal until within a year, when it had become profuse; the first of the flow was "muddy" and the last colorless. For two years she had had longing for unnutritious articles. At first she ate charcoal, afterward, fine sand. She would eat three or four tablespoonfuls in a day, and hardly a day passed when she did not eat some of it. She declared she had eaten "nearly a bushel" of the sand.

She had gained flesh while eating this substance, although she had some dyspnœa on going up stairs, had pale lips, and was constipated. When supplied with carbonate of iron to eat, she did not crave the sand.

